# An efficient strategy for fine-tuning large language models

**DOI:** 10.3389/frai.2026.1665992

**Published:** 2026-02-17

**Authors:** Benjamin Marsh, Adam Michaleas, Darrell O. Ricke, Shaun Monera, Shriya Zembruski

**Affiliations:** 1 Marine Corps Tactical Systems Support Activity, United States Marine Corps, Camp Pendleton, CA, United States; 2 MIT Lincoln Laboratory, Artificial Intelligence Technology, Lexington, MA, United States

**Keywords:** deep learning, distributed computing, fine-tuning, large language models, neural networks, NLP

## Abstract

**Introduction:**

Large Language Models (LLMs) achieve strong performance on many Natural Language Processing tasks, but adapting them to domain-specific applications is resource-intensive due to the cost of curating task-specific datasets and the compute required for fine-tuning. This work proposes an end-to-end strategy for rapidly fine-tuning LLMs for domain-specific tasks when both data and compute are limited.

**Methods:**

The strategy uses Distilling Step-by-Step (DSS) for dataset development and model training, where a teacher model generates task labels and intermediate rationales via Chain-of-Thought prompting for a natural-language-to-Query-DSL structured generation task. Using the resulting supervision, we benchmark three fine-tuning modalities through hyperparameter sweeps: full-precision fine-tuning, Low-Rank Adaptation (LoRA), and Quantized LoRA (QLoRA). To isolate the effect of rationale supervision, we additionally conduct an ablation study comparing DSS training (label + rationale supervision) against a label-only configuration.

**Results:**

Across the evaluated configurations, DSS combined with full-precision fine-tuning yields the strongest overall performance. Under resource constraints, DSS with LoRA provides an effective performance-efficiency tradeoff, and DSS with QLoRA enables training under tighter GPU memory budgets while maintaining competitive performance. In the parameter-efficient regimes, an alpha-to-rank ratio of 4:1 provides a consistent balance of performance and compute consumption across the explored settings.

**Discussion:**

These findings support a practical process for resource-constrained domain adaptation: use DSS to efficiently construct datasets, then select the fine-tuning modality based on available compute (full-precision when feasible; LoRA or QLoRA when memory-limited). The proposed workflow offers a general guide for efficiently fine-tuning LLMs for domain-specific tasks with limited data availability.

## 1 Introduction

Large Language Models (LLMs) are state-of-the-art NLP tools based on the transformer neural network architecture (Vaswani et al., 2023). Classical Natural Language Processing (NLP) methods rely on mechanical translation and statistical methods for text prediction (Bengio et al., 2000). Classical fine-tuning methods for LLMs require significant resources and time to develop datasets and to execute the fine-tuning procedure. This paper introduces an approach to developing datasets and fine-tuning LLMs for domain-specific tasks with limited datasets in resource-constrained compute environments, namely, applying Distilling Step-by-Step (DSS) for dataset development in a distributed computing regime. This approach is then used as a base for a comparative analysis of established fine-tuning methods to determine the most efficient fine-tuning method for rapidly developing models for domain-specific tasks.

Although LLMs have been repeatedly shown to have excellent performance on text-based tasks, there are limitations that constrain their application for specialized use cases. One limitation is that LLMs are trained using large datasets that are composed of general-purpose text and often do not contain information on specialized domains such as law or medicine (Nori et al., 2023). This lack of specialized domain knowledge often results in LLM-generated text that appears coherent but is incorrect (Zhang et al., 2023). LLMs often consist of hundreds of millions to hundreds of billions of parameters. The sheer size of these models requires the use of systems with large amounts of compute capacity, which incurs a major financial cost for both the on-premises and cloud computing usage paradigms (Chen et al., 2023).

Fine-tuning and related strategies remain a standard approach for aligning pretrained LLMs to specialized tasks and constraints. We summarize the relevant literature on fine-tuning in Section 2. In this study, we benchmark full-precision fine-tuning against parameter-efficient alternatives, Low Rank Adaptation (LoRA) and Quantized Low Rank Adaptation (QLoRA) under GPU memory constraints consistent with limited compute environments. To reduce the cost of dataset development, we use Distillation Step-by-Step (DSS) (Hsieh et al., 2023) to construct a task-specific data set that contains both output labels and intermediate rationales, and we explicitly ablate rationale supervision versus label-only training. We use FLAN-T5 (Small/Base/Large/XL) as an instruction-tuned sequence-to-sequence translation exemplar to study scaling behavior across fine-tuning modalities on a structured-generation task. FLAN-T5 is selected due to its strong performance on translation tasks and its suitability for text-to-structured generation tasks (Wei et al., 2022).

In this work, we detail the extension of the DSS method to the FLAN-T5 language models and benchmark DSS performance across standard fine-tuning, LoRA, and QLoRA fine-tuning methods while using the novel dataset created via DSS: natural language questions to Query Domain-Specific Language (DSL). Additionally, while the DSS method provides an efficient mechanism for developing fine-tuning datasets that include both labels and intermediate reasoning chains, the contribution of rationales relative to label-only supervision remains an open methodological question. Previous research has shown that DSS improves model learning efficiency on open-source benchmark datasets (Hsieh et al., 2023). However, a systematic comparison of DSS fine-tuning versus label-only fine-tuning has not been reported on task-specific fine-tuning datasets, especially in the context of parameter-efficient fine-tuning approaches and quantized optimization regimes.

To address this gap, this study includes an ablation experiment designed to isolate the effect of rationale supervision by comparing DSS against a label-only training configuration. The ablation spans multiple model sizes and fine-tuning methods to assess whether the contribution of rationales varies with model capacity or training constraints.

Large language models are a marked advance in the fields of deep learning and natural language processing. In this work, we emphasize the importance of efficiently fine-tuning these models for domain-specific tasks that fall outside of their original training data. This work provides a guide for efficiently fine-tuning LLMs for domain-specific tasks with limited data availability. DSS combined with full-precision fine-tuning provided better results. For resource-constrained environments, use Low Rank Adaptation fine-tuning with the Alpha to Rank ratio of 4:1 to balance performance and computation consumption. For GPU-limited environments, use Quantized Low Rank Adaptation fine-tuning with an Alpha to Rank ratio of 4:1. The code and instructions are available at the following Git Repository link: https://github.com/brmarsh23/An-Efficient-Strategy-for-Fine-Tuning-Large-Language-Models.

## 2 Related work

### 2.1 Domain adaptation and instruction-tuned sequence-to-sequence models

**Figure 1:**
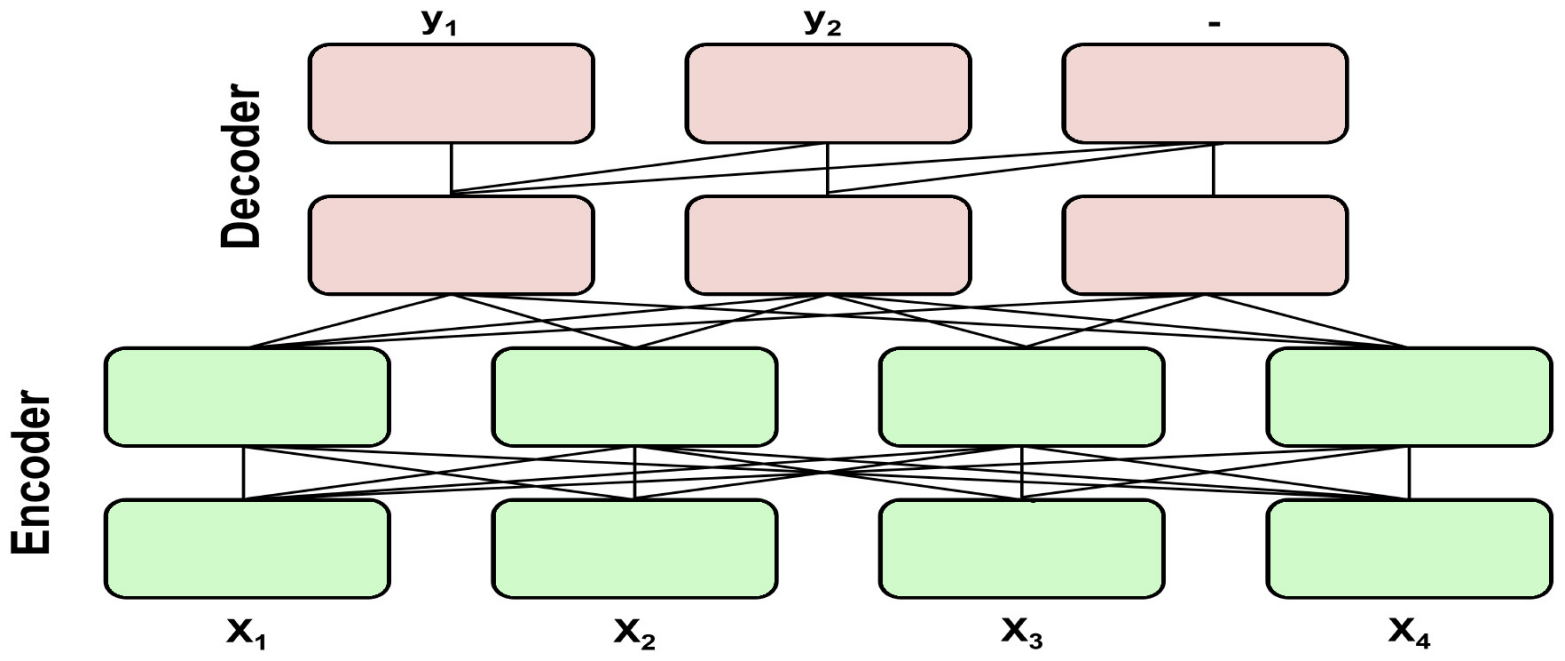
Encoder-decoder architecture adapted from Raffel et al. (2023, Figure 4, CC4.0 changes: redrawn). T5 models use an encoder-decoder architecture with all input tokens fully visible in the encoder and encoder-decoder attention, and causal masking in the decoder. The encoder consists of identical layers of self-attention mechanisms, feedforward networks, and layer normalizations. The decoder consists of identical layers of self-attention mechanisms, feedforward networks, layer normalizations, and causal masking.

Modern LLMs are predominantly transformer-based (Vaswani et al., 2023) and achieve strong performance on general-purpose tasks via pretraining on large datasets. The text-to-text framing method introduced by the T5 family of encoder-decoder models (Figure 1) provides a sequence-to-sequence translation process for a wide range of downstream tasks (Raffel et al., 2023). Building on this foundation, instruction tuning has been shown to substantially improve zero-shot generalization for T5 models, including FLAN-T5 (Wei et al., 2022). These instruction-tuned sequence-to-sequence models are therefore a practical starting point for domain-specific text generation tasks, particularly when the target can be expressed as a structured mapping from an input text prompt to an output text sequence.

However, general-purpose pretraining does not guarantee reliable performance in specialized domains. Empirical studies have documented failures such as confident but incorrect model outputs, described as hallucinations (Zhang et al., 2023), and poor performance on domain-specific tasks when relevant knowledge is underrepresented in pretraining data (Nori et al., 2023). More broadly, domain and task mismatch has motivated continued emphasis on model adaptation methods, including domain-specific pretraining and fine-tuning, as ways to align model behavior with a target downstream task (Gururangan et al., 2020; Tinn et al., 2023).

### 2.2 Full-precision and parameter-efficient fine-tuning

Full-precision fine-tuning adapts a pretrained model by updating a large fraction of parameters, often yielding strong downstream performance but incurring substantial computational and memory costs for transformer-scale models. These compute and memory costs arise from storing model parameters, gradients, and optimizer states and the distributed training overhead that scales with model size and sequence length. Large-scale optimization techniques such as ZeRO reduce memory redundancy and improve feasibility for large model training, but practical constraints remain for resource-limited environments (Rajbhandari et al., 2020).

Parameter-efficient fine-tuning (PEFT) approaches reduce fine-tuning compute and memory costs by restricting trainable parameters. PEFT approaches include LoRA (Low-Rank Adaptation) for injecting low-rank matrices, adapters for adding small bottleneck layers, prefix tuning/prompt tuning for learning continuous prompts, and QLoRA/DoRA for enhanced efficiency (quantization/decomposition). Herein, the PEFT methods LoRA and QLoRA are compared with full-precision fine-tuning. LoRA injects low-rank trainable adapter weight matrices into targeted model layers while freezing the base weights, enabling competitive downstream performance with fewer trainable parameters than the full-precision method (Hu et al., 2021). QLoRA extends this regime by quantizing the base model weights while training adapters weight matrices, improving memory efficiency and enabling fine-tuning of larger models under higher compute constraints (Dettmers et al., 2023). Surveys and unifying perspectives on PEFT further emphasize that adapter-based methods can provide strong performance-efficiency tradeoffs across tasks and model classes (Han et al., 2024; He et al., 2022).

### 2.3 Knowledge distillation, chain-of-thought, and rationale supervision

Knowledge distillation is the transferring of capability from a larger teacher model to a smaller student model by using teacher-produced targets as supervision for the student (Hinton et al., 2015). Distillation Step-by-Step (DSS) augments this paradigm by training on both the targets and intermediate rationales produced by the teacher model, typically elicited through chain-of-thought prompting (Wei et al., 2023; Hsieh et al., 2023). DSS has been shown to improve data efficiency on benchmark reasoning tasks, enabling smaller models to approach or exceed the performance of larger models trained with conventional supervision under comparable compute and dataset constraints (Hsieh et al., 2023).

However, the extent to which rationale supervision improves downstream performance may be task dependent. This open question motivates ablation studies that compare rationale-augmented training against label-only supervision under controlled training conditions, especially when combined with PEFT and quantized optimization regimes where model capacity and optimization dynamics differ from full-precision training (Hu et al., 2021; Dettmers et al., 2023).

### 2.4 Natural language to structured query generation

Mapping natural language questions to executable structured queries is a classic semantic parsing problem, widely studied in text-to-SQL and related benchmarks. Large-scale datasets such as Spider emphasize cross-domain generalization and complex compositional query generation (Yu et al., 2018), while earlier approaches established sequence-to-sequence structured query generation as a viable modeling strategy (Zhong et al., 2017). A persistent practical challenge in this area is output validity: syntactically invalid structured outputs are unusable even when semantically close to the target. Constrained decoding methods such as PICARD explicitly enforce admissible structure during autoregressive decoding and have been shown to improve validity and performance with models such as T5 (Scholak et al., 2021).

In operational systems, structured query representations also extend beyond SQL to domain-specific query languages. Query DSL, as used in OpenSearch, is one such representation and is commonly expressed as JSON (Contributors, 2024). This motivates evaluation setups that consider both semantic correctness and structural validity when fine-tuning LLMs for natural-language to Query-DSL translation.

### 2.5 Positioning of this work

Building on instruction-tuned sequence-to-sequence modeling (Wei et al., 2022; Raffel et al., 2023), PEFT methods (LoRA/QLoRA) (Hu et al., 2021; Dettmers et al., 2023), and DSS rationale-augmented fine-tuning (Hsieh et al., 2023), this work proposes and evaluates a strategy for constructing a domain-specific dataset via DSS and benchmarks full-precision fine-tuning against LoRA and QLoRA under realistic resource constraints. Additionally, we include an ablation study comparing rationale-augmented training to label-only supervision to isolate the contribution of rationale supervision within this task setting.

## 3 Materials and methods

### 3.1 Distilling step-by-step

Distilling Step-by-Step is a method by which a teacher LLM is used to generate feature labels and label rationales in order to train a smaller model to match the teacher model's performance on the task. DSS fine-tuned models have been shown to achieve superior performance, using smaller fine-tuning datasets, than larger models trained with standard fine-tuning methods on benchmark datasets and on few-shot learning tasks (Hsieh et al., 2023).

**Figure 2:**
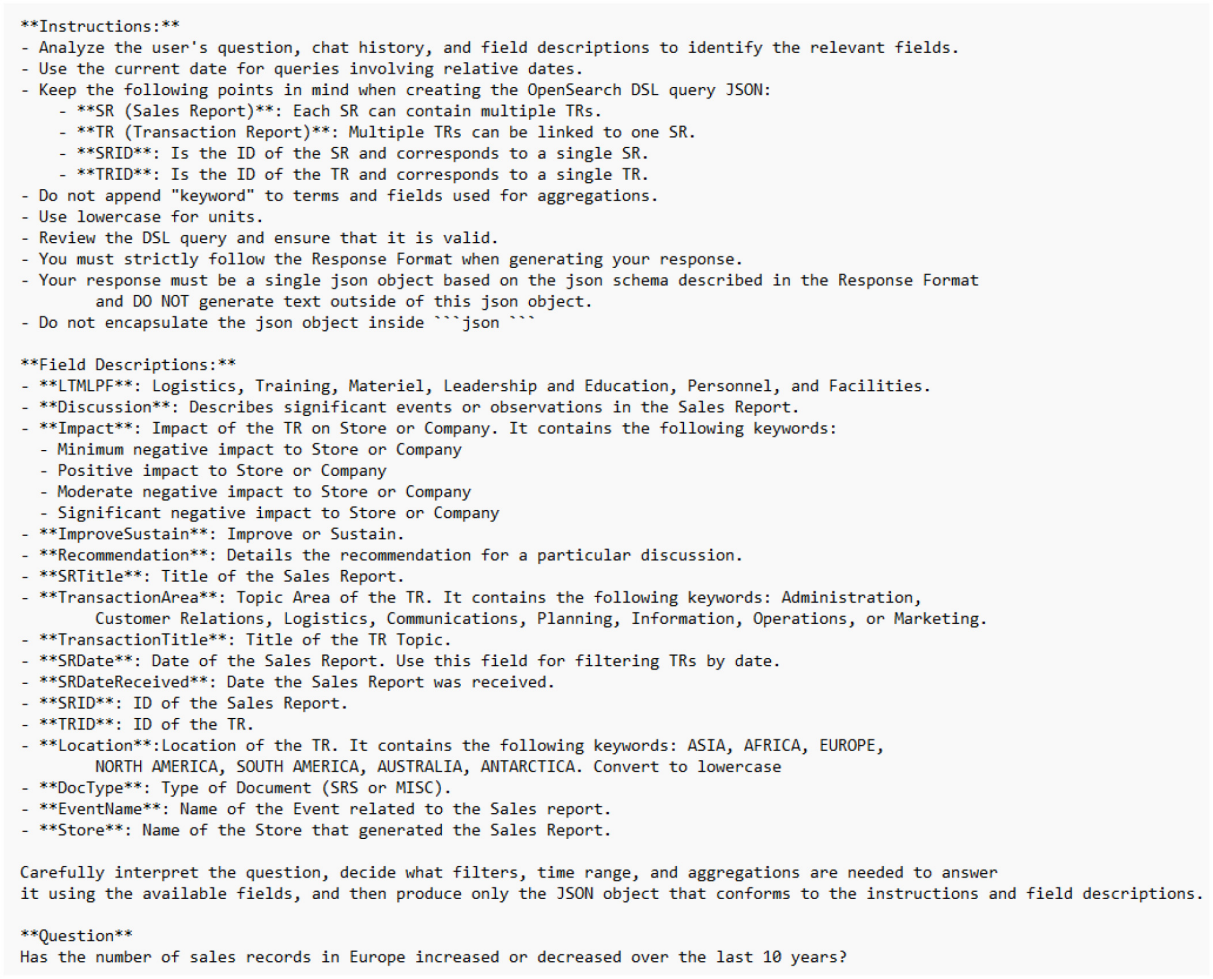
Example input prompt. The prompt contains instructions for properly formatting DSL queries, descriptions of the downstream task dataset, and Chain-of-Thought prompting.

The generation of label rationales was done by prompting teacher models using chain-of-thought (Wei et al., 2023). This technique provided intermediate rationales to explain the connections between the input and the label (Hsieh et al., 2023). An example input prompt is shown in Figure 2. The prompt consists of three distinct parts that are designed to provide information on the DSL interface and dataset of the downstream task while inducing a chain-of-thought reasoning process in the model. The final section, **Question**, contains an example input question from the user. Using this prompt schema, the rationals and DSL JSON output labels were extracted from the teacher model.

Once rationales and labels were extracted for all inputs, the rationales were incorporated into the fine-tuning dataset by setting up the model fine-tuning as a multi-task problem. For each input, a task prefix was prepended to the input features. Each task prefix corresponds to the resulting output (label or rationale) and enables the student model to differentiate between each task (label generation or rationale generation). Thus, the student model was trained to generate the correct rationales and labels for each input.

### 3.2 Dataset description

**Figure 3:**
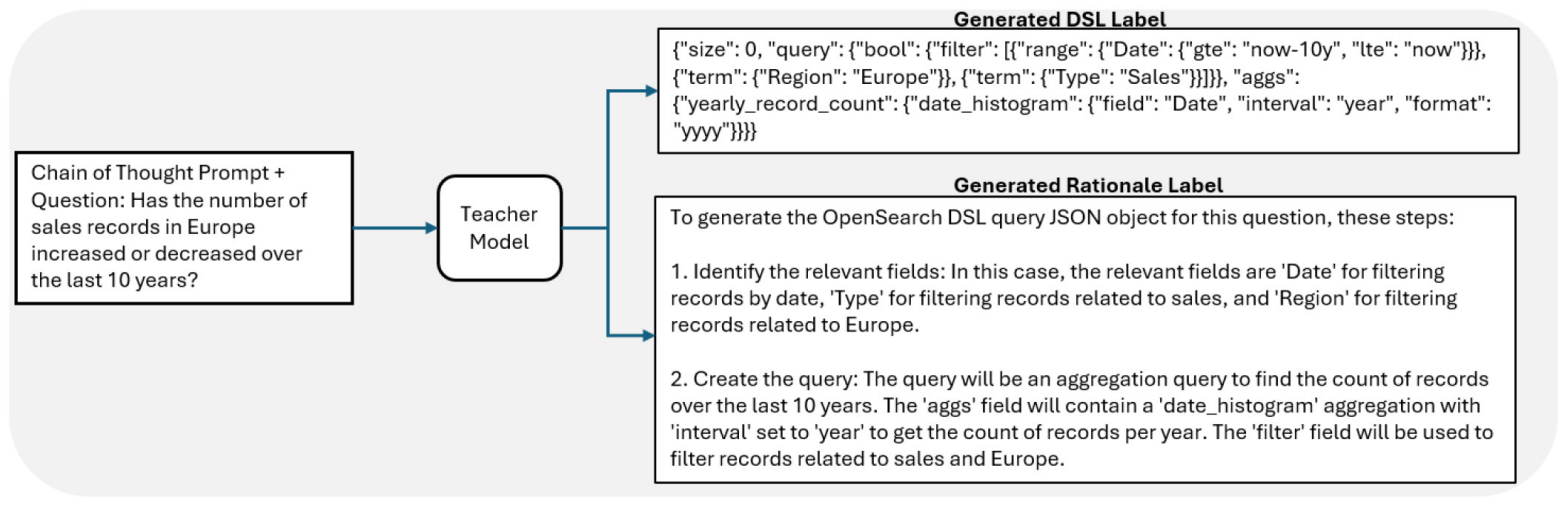
Overview of dataset creation using DSS. Chain-of-Thought prompting is used to extract rationales and labels from the teacher model. In this work, the Mixtral 8x22B LLM was used as the teacher model.

**Figure 4:**
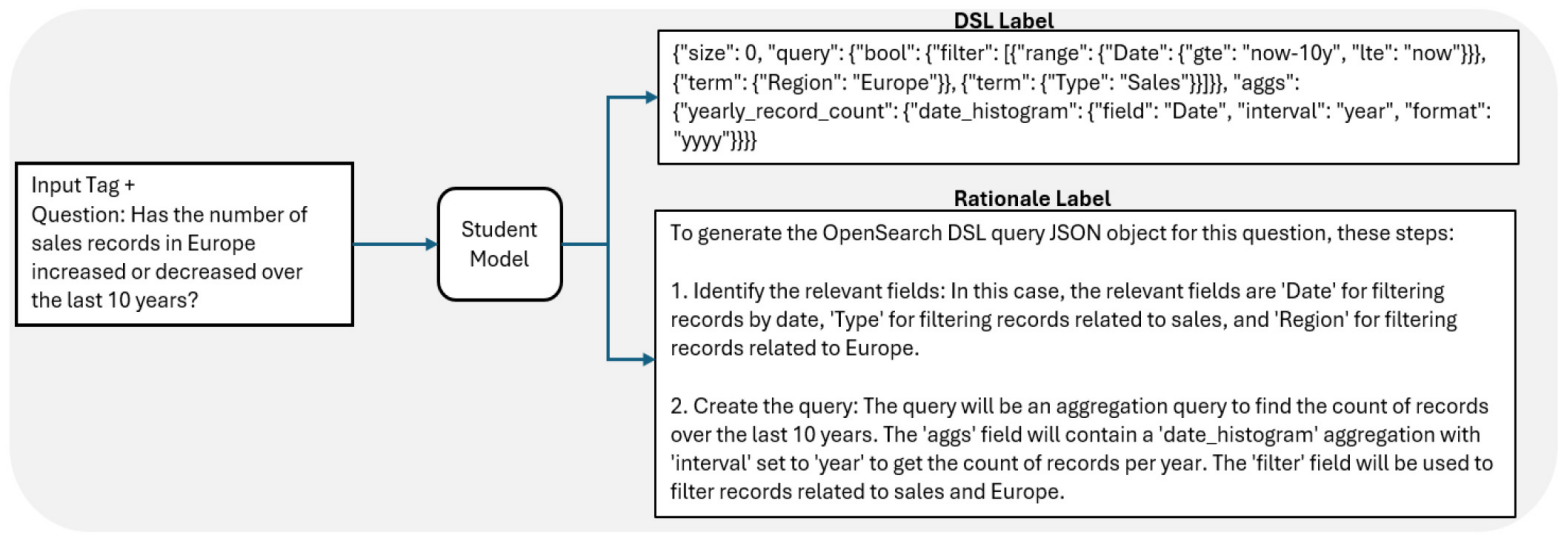
Overview of an example fine-tuning training step using a dataset generated by DSS. An input tag (rationale or label) was pre-pended to the input, and the student model was trained to generate the label that matches the input tag.

This work utilized a fine-tuning dataset designed around the conversion of natural language questions to Query Domain-Specific Language, or Query DSL, for use by practitioners to query organizational data without requiring knowledge of database querying languages. Fine-tuning a language model to perform this translation task would enable practitioners to interact with databases in a more conversational way, leading to broader use. Query DSL is a flexible search language used by the OpenSearch data-analysis platform to parse through databases (Contributors, 2024). The fine-tuning dataset input features consisted of 1000 natural language questions to be translated to Query DSL for searching through the OpenSearch database. Each of these input questions was matched to an output label that consisted of a properly formatted Query DSL JavaScript Object Notation (JSON) object that provided the desired OpenSearch results based upon the input question and an output rationale that described how to generate the corresponding Query DSL JSON in a concise, step-by-step fashion. Figure 3 gives an example rationale and label generation process for a given input. Figure 4 shows an example training step using labels and rationales. Both the Query DSL output labels and rationales were generated as described in the previous subsection, using the open-source model Mixtral 8x22B, a Sparse Mixture of Experts (MOE) LLM (Team, 2024) as the teacher model. In total, the dataset consisted of 1000 input questions, along with 1000 output labels and rationale pairs. The full dataset was used for fine-tuning.

### 3.3 Model architecture

The multiple architectures of the FLAN T5 model that were used in this work are shown in Table 1. There are several key differences between each model variant. Most notably, the total number of parameters is governed by the number of encoder and decoder layers, the dimensionality (i.e., size) of the feedforward layers, and the number of attention heads per attention block within each encoder and decoder layer. Between the FLAN T5 Small, T5 Base, and T5 Large, there is a consistent increase in the dimensionality of the feedforward layers, the number of encoder and decoder layers, and the number of attention heads, which results in a consistent increase in the total number of parameters as the model size increases. However, the FLAN T5 XL has feedforward layers with four times as many parameters as the FLAN T5 Large and twice as many attention heads per block, resulting in a large increase in the total number of parameters in the FLAN T5 XL over the FLAN T5 Large. In this work, we utilized FLAN T5 Small, Base, Large, and XL architectures to determine the performance and efficiency of the three fine-tuning strategies (full-precision, LoRA, and QLoRA) using the Query DSL dataset.

**Table 1:** FLAN T5 model variants used in this work.

**Model**	**Parameters**	**Layers**	**Size**	**Heads**
FLAN T5 Small	76.956	6	2,048	8
FLAN T5 Base	247.577	12	3,072	12
FLAN T5 Large	770.567	24	4,096	16
FLAN T5 XL	2,884.497	24	16,384	32

Model size is measured in millions of parameters. Layers column gives the number of encoder and decoder layers per model. Linear size gives the dimension of the linear layers. Number of Attention Heads are given per attention block.

### 3.4 Fine-tuning methods

**Figure 5:**
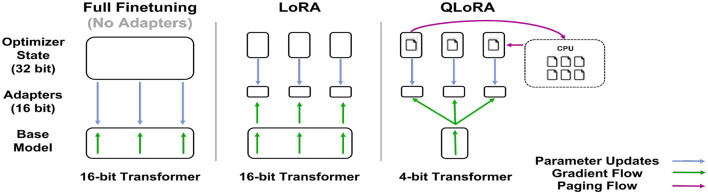
Comparison of fine-tuning methods from Dettmers et al. (2023, Figure 1, CC4.0). Full-precision fine-tuning directly updates the model weights. LoRA fine-tuning trains rank decomposition weight matrices while holding full-precision model weights constant. QLoRA quantizes the full-precision model weights and utilizes memory-ecient paged optimizers.

Fine-tuning is the process by which a pre-trained language model is trained on additional tasks using a smaller dataset than the pre-training corpus. The classical fine-tuning method is to make some or all of the language model parameters available for training using full floating-point precision model weights, then train the model weights using a lower learning rate than the learning rate used during pre-training. In this work, this classical fine-tuning method is referred to as full-precision fine-tuning. Although the full-precision method can be very effective in terms of performance, it comes at a high computational cost, especially in terms of GPU memory consumption (Quentin and Stella Biderman, 2023). Due to this high cost, efficient methods have been developed to provide a balance between cost and performance. Two such methods, along with full-precision fine-tuning, were investigated: LoRA and QLoRA fine-tuning (see Figure 5).

LoRA fine-tuning, originally demonstrated in Hu et al. (2021), freezes the pre-trained model weights and injects trainable rank decomposition matrices into specified layers of the transformer architecture, which, due to reducing the number of trainable parameters for downstream tasks, theoretically reduces the computational cost of fine-tuning the model. As shown in Hu et al. (2021), a GPT-3 175B model fine-tuned with the Adam optimizer and LoRA fine-tuning reduced the number of trainable parameters by 10,000 times and the GPU memory requirements by 3 times while performing on par or better than standard fine-tuning on several benchmarks (Hu et al., 2021).

QLoRA fine-tuning is designed for further reductions in computational cost without sacrificing model performance. Originally shown in Dettmers et al. (2023), QLoRA quantizes the pre-trained model weights to 4-bit NormalFloat, a theoretically optimal quantization data type (Dettmers et al., 2023) and utilizes paged optimizers to avoid memory spikes that occur when processing mini-batches with long sequence lengths (Dettmers et al., 2023). QLoRA has been shown to match full-precision fine-tuning and LoRA fine-tuning performance on multiple benchmarks (Dettmers et al., 2023).

### 3.5 Distributed computing and training

To provide scalable distributed computing capabilities for model training, a two-node, GPU-enabled Rancher 2.0 Community Edition (Rancher, 2024) Kubernetes cluster was utilized for the hyperparameter search. The compute nodes were provisioned with two Intel Xeon Platinum 8480+ processors, 2 TB RAM, four NVIDIA 80 GB H100 GPUs, and non-volatile memory express (NVME) solid-state storage.

Each variant of FLAN T5 was trained on the Query DSL dataset developed using DSS. Fine-tuning jobs were submitted using the Ray Train configured for Distributed Data Processing through the Accelerator API (Team, 2023).

The fine-tuning dataset was split in an 80/20 fashion into training and evaluation datasets, and all hyperparameter sweeps utilized the same split. The models were trained for 100 epochs with evaluation taking place after each epoch, and the evaluation loss was monitored in order to conduct learning rate reduction after 10 epochs of no improvement. Loss was calculated in accordance with (Hsieh et al. 2023):


$$\begin{array}{l} L_{label}=\frac{1}{N}\overset{N}{\underset{i=1}{\sum }}l\left(f\left(x_{i}\right),y_{i}\right) \end{array}$$ (1)

where *L*_*label*_ is the label prediction loss and *l* is the cross-entropy loss between the predicted tokens *x*_*i*_ and label tokens *y*_*i*_. Additionally, we framed the learning as a multi-task problem in accordance with Hsieh et al. (2023), where we train the model to predict not only the task labels but also the rationales given an input:


$$\begin{array}{l} L_{total}=\alpha \left(L_{label}\right)+\left(1-\alpha \right)\left(L_{rationale}\right) \end{array}$$ (2)

where *L*_*total*_ is the combined loss between *L*_*label*_, described above, with *L*_*rationale*_


$$\begin{array}{l} L_{rationale}=\frac{1}{N}\overset{N}{\underset{i=1}{\sum }}l\left(f\left(x_{i}\right),r_{i}\right) \end{array}$$ (3)

this is the cross-entropy loss between the predicted tokens *x*_*i*_ and the rationale for the label *r*_*i*_. In Equation 2, α was used as an additional training hyperparameter to tune how much loss the model accumulates from predicting rationales. For this work, α was set to 0.5 to balance the loss between predicted rationales and predicted labels.

### 3.6 Hyperparameter search

The hyperparameters that were utilized across all of the hyperparameter sweeps are shown in Table 2. The learning rate was based upon optimal results found in Hsieh et al. (2023) for the T5 neural network architectures, and the batch size was given as the number of training samples per batch across all nodes. In this work, one training sample per batch for each of the 8 nodes in the cluster results in a total batch size of 8 training samples per batch. As shown in Equation 2, Alpha was set to 0.5 to balance the loss resulting from rationales and labels.

**Table 2:** Hyperparameters that were utilized across all of the fine-tuning runs, excluding model architecture.

**Hyperparameter**	**Value**
Learning rate	5e-5
Learning rate patience	10 epochs
Learning rate factor	1e-1
Number of epochs	100
Total batch size	8
Alpha	0.5

Total batch size is given as the number of training samples per batch across all nodes.

Selected LoRA and QLoRA-specific hyperparameters (Target Modules and Dropout Rate) were held consistent throughout the hyperparameter sweeps for both methods. The target modules were selected based upon performance detailed in Dettmers et al. (2023), and dropout rate (Srivastava et al., 2014) was selected based upon performance considerations given in Lin et al. (2024). The other hyperparameter spaces searched during this work were the LoRA and QLoRA Rank and Alpha hyperparameters. The Rank hyperparameter determines the dimensionality of the LoRA/QLoRA matrices (Hu et al., 2021), and the Alpha hyperparameter serves as a scaling factor for the updates made by the LoRA/QLoRA matrices to the original model weights (Hu et al., 2021). For a given value of Rank, a series of Alpha hyperparameters were tested. The Rank values tested in this work were 32, 64, and 128. Alpha values paired with Rank 32 were 32, 64, and 128. Alpha values paired with Rank 64 were 32, 64, 128, and 256. Alpha values paired with Rank 128 were 32, 64, 128, 256, and 512. In this way, Alpha to Rank ratios of 1:4, 1:2, 1:1, 2:1, 3:1, and 4:1 were represented in the hyperparameter search to determine if an optimal Alpha to Rank ratio exists.

The hyperparameter search consisted of 86 hyperparameter sweeps spread across all three methods and for the four model types examined. For a given set of hyperparameters, a fine-tuning run for each model architecture and fine-tuning method was attempted, beginning with the smallest model. If the run was successful, the next model architecture, in order of increasing size, was utilized for the next run attempt. This process was continued for each successive model architecture until the models would not run due to GPU memory limitations on the cluster. After this failure point was reached, the next fine-tuning method would be selected, and the process would be repeated with the same hyperparameters, beginning with the smallest model. When all fine-tuning methods and model architecture sizes were attempted for a given set of hyperparameters, the hyperparameter set would be changed, and the entire process would begin anew. In total, 3 runs were performed using full-precision fine-tuning, 39 runs were performed with the LoRA fine-tuning method, and 44 runs with the QLoRA fine-tuning method. Twenty-nine hyperparameter sweeps were performed using the FLAN T5 Small model architecture, 29 using the FLAN T5 Base architecture, 29 using the FLAN T5 Large architecture, and 5 sweeps using the FLAN T5 XL architecture. Due to GPU memory usage constraints, all of the FLAN T5 XL runs were executed using the QLoRA fine-tuning method. The total compute time for the hyperparameter search was 499.6 hours.

### 3.7 Ablation study design

To quantify the contribution of DSS rationales to the performance of the downstream task, we performed an ablation comparing two variants of the loss function described in Equation 2: rational-augmented supervision (Alpha = 0.5), where both label and rationale predictions contribute equally to the total loss, and label-only supervision (Alpha = 1.0), where loss due to rationales is ignored during optimization. All other hyperparameters were kept constant throughout to ensure that differences in performance could be attributed exclusively to the presence or absence of rationale supervision.

The ablation spanned all three fine-tuning modalities and three model sizes: FLAN-T5 Small, Base, and Large. These models were selected to capture the variation in the model size while allowing all modalities to be compared, given the computational constraints. Each ablation run used the same data set and train-validation split as the full experiments, and the results were averaged over two random seeds to reduce the variance due to initialization effects.

### 3.8 Evaluation strategy and metrics

For the final evaluation, the best evaluation loss achieved for each hyperparameter sweep was used to perform the final comparison. The fine-tuned student models across all modalities were tested using the same evaluation data. Additionally, the computational cost of each fine-tuning method was compared by using training samples per second and total training time metrics for each model and fine-tuning modality that were captured by the Rancher Kubernetes Cluster.

## 4 Results

**Figure 6:**
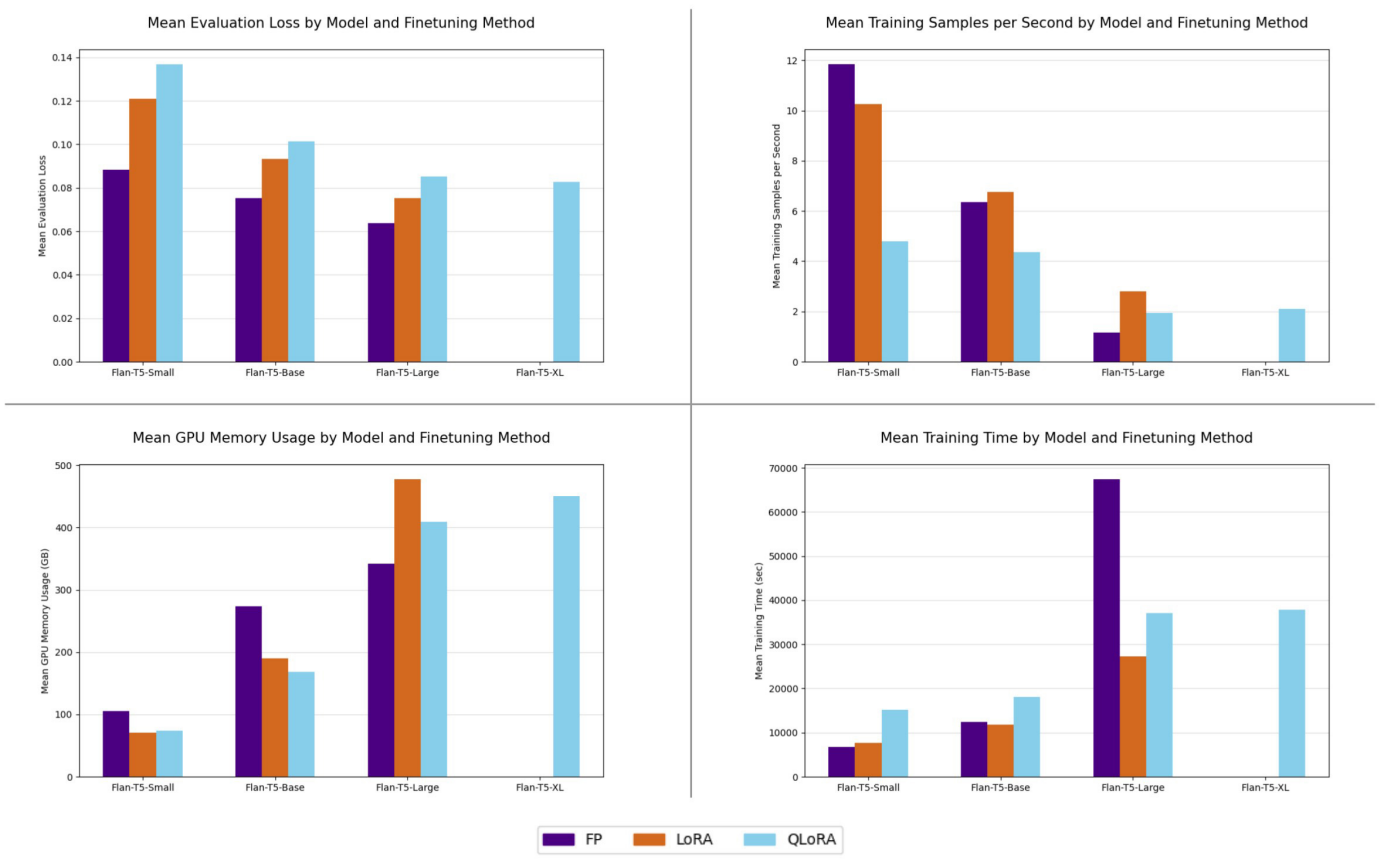
Quad chart details the average computational cost per model for each fine-tuning method, along with the mean evaluation loss per model for each fine-tuning method.

First, the average metrics of the hyperparameter search for each model and fine-tuning method are shown in Figure 6. The bottom left chart shows the mean GPU memory usage per model type and fine-tuning method. For the FLAN-T5 Small and FLAN-T5 Base model types, the full precision method utilized the most memory on average, followed by the LoRA and QLoRA methods. For the FLAN-T5 Large model type, the full-precision method uses, on average, the least amount of GPU memory, followed by the QLoRA method. The LoRA method, on average, used the most GPU memory. The average memory usage in each fine-tuning method increased with increasing model size, but a marked difference was observed in the rate of increase of memory usage between the LoRA and QLoRA methods and the full precision method. The full-precision method had a relatively small increase in memory usage between the FLAN-T5 Base and FLAN-T5 Large model types, whereas the LoRA and QLoRA methods demonstrated over twice the average memory consumption during the FLAN-T5 Large model runs over the FLAN-T5 Base model runs.

Next, computational metrics aside from GPU memory consumption are shown in the top right chart in Figure 6; this chart shows the average training samples per second for each model and fine-tuning method. The training samples per second metric represents the total number of training samples processed across all workers in the cluster per second, not per individual worker. The full-precision method demonstrates the highest average training samples per second with the FLAN-T5 Small architecture, with the LoRA and QLoRA methods demonstrating the second-highest and third-highest averages, respectively. All fine-tuning methods demonstrate reduced training samples per second averages as the model architectures increase in size, but the full-precision method experiences the largest drop across architectures. The LoRA method demonstrates the highest average training samples per second with the FLAN-T5 Base, while the full-precision method comes in second, and QLoRA in third. For the FLAN-T5 Large model architecture, the full-precision method demonstrates the lowest average training samples per second, while LoRA has the highest, and QLoRA is in the middle. Notably, the FLAN-T5 XL model run with the QLoRA method demonstrates a small improvement in average training samples per second over the FLAN-T5 Large model runs. While average training samples per second are useful for showing a close look at training efficiency, the average total training time for an individual hyperparameter sweep gives an estimate of the total computational cost of training a model.

Third, the average total time of a hyperparameter sweep is shown in Figure 6 bottom right chart; this chart compares the average total time of a hyperparameter sweep, given in seconds, for each model architecture and fine-tuning method. The full-precision model method demonstrates a rapid increase in average total training time between the FLAN-T5 Small, Base and Large model architectures, going from the fastest method on average from the FLAN-T5 Small, to the second-fastest with FLAN-T5 Base, to the slowest method with FLAN-T5 Large. The LoRA and QLoRA methods, by comparison, demonstrate smaller, more consistent increases in average training time. Both methods demonstrate, roughly, a doubling of average training time between the FLAN-T5 Base and FLAN-T5 Large architectures. The QLoRA method shows a minor increase in average training time between the FLAN-T5 Large and FLAN-T5 XL architectures. The observation that the LoRA and QLoRA methods, on average, required more time to train than the full-precision method on smaller model sizes can be explained by the additional overhead required by these methods to compute forward and backward passes on the adapter matrices and, for QLoRA, to dequantize the 4-bit model during forward and backward passes. The full-precision method does not require this additional overhead, but the overall cost of tuning the full model leads to greater training times at the larger model sizes.

Fourth, the average evaluation loss per model architecture and fine-tuning method is shown in Figure 6, top left chart. These averages take into account the entire hyperparameter search for each model architecture and fine-tuning method. The fine-tuning methods are consistent in their performance ranking across all of the model architectures. The full-precision fine-tuned models all perform better, on average, than the LoRA and QLoRA fine-tuned models, with the QLoRA fine-tuned models performing, on average, worse than the LoRA fine-tuned models. Observing all four charts together, the full-precision method, on average, has better performance and faster training times at the cost of higher GPU memory usage than the other fine-tuning methods for the FLAN-T5 Small and FLAN-T5 Base model architectures.

For the FLAN-T5 Large architecture, however, the full-precision fine-tuning method used less GPU memory, on average, than the LoRA and QLoRA methods. Although this finding may seem counterintuitive given the theoretical memory efficiencies of the LoRA and QLoRA methods, the result aligns with previous research (He et al., 2022). Specifically, the implementation of these methods introduces additional memory overhead in several different ways. First, while full-precision only updates the original model parameters, LoRA adds extra trainable matrices that, while small in comparison to the size of the original model, introduce additional parameters and optimizer states that must be stored in memory. This memory penalty increases as the number of layers in the original model increases. In this case, there are twice as many layers in the FLAN T5 Large and XL models as in the FLAN T5 Base model (see Table 1). These LoRA parameters and optimizer states are held in memory in addition to the original model during forward and backward passes, leading to additional memory consumption over full-precision fine-tuning. Next, the QLoRA method, while using a quantized original model during the training process, requires that the 4-bit model weights be dequantized to half-precision (FP16) during forward and backward passes (Dettmers et al., 2023), which, when combined with the aforementioned LoRA penalty, may help explain the increased memory consumption for the FLAN T5 Large and XL model sizes. Additionally, implementation differences between the full-precision and LoRA/QLoRA methods are another potential source of additional memory consumption. Full-precision utilizes standard PyTorch libraries (PyTorchFoundation, 2025) for training, whereas the other methods utilize Hugging Face Parameter-Efficient Fine-Tuning (PEFT) (HuggingFace, 2025b) and bitsandbytes (HuggingFace, 2025a) libraries, which may not be optimized for memory in the same way as the standard PyTorch libraries.

**Figure 7:**
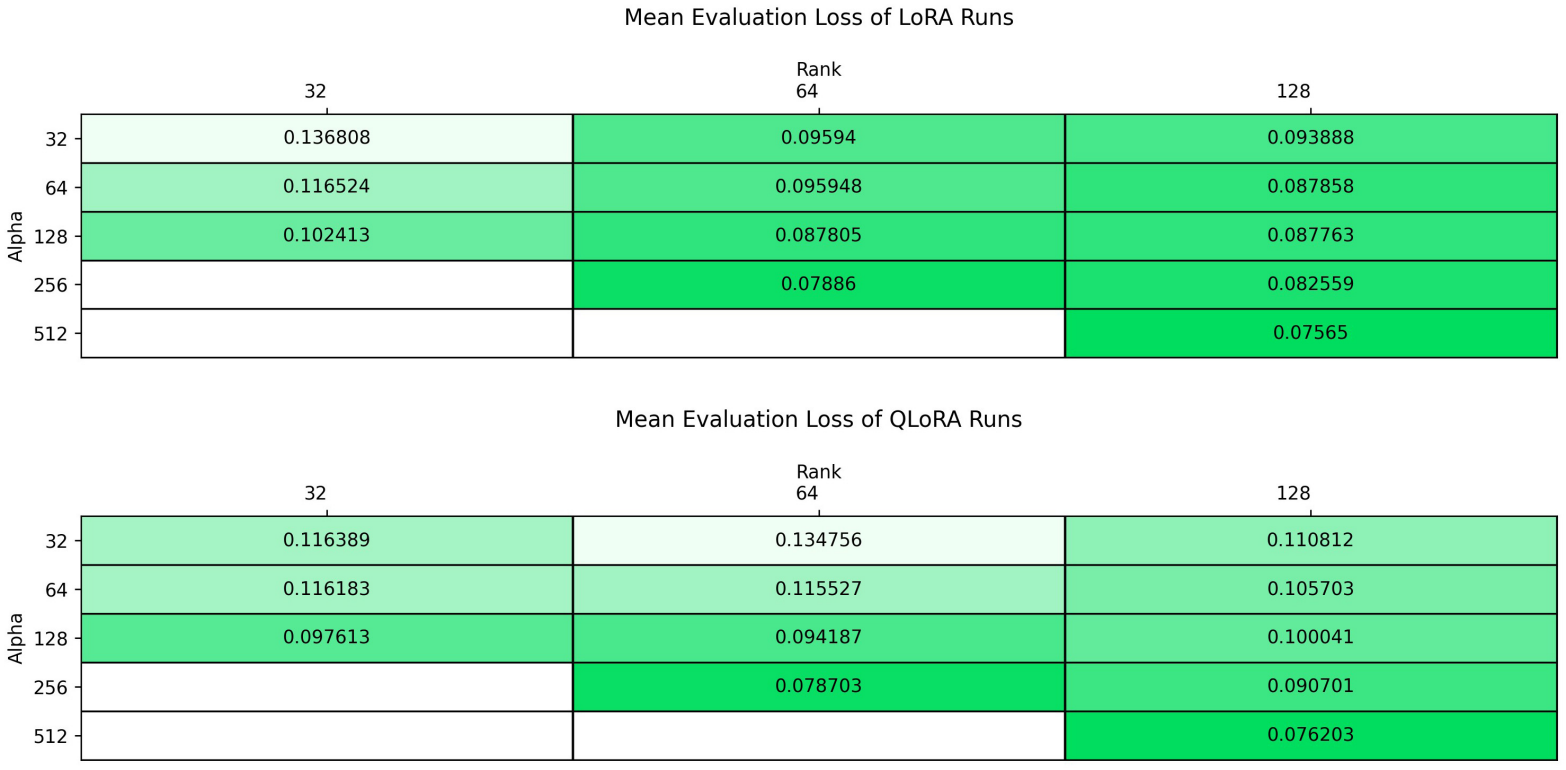
Average evaluation loss of each rank and alpha combination for the LoRA and QLoRA fine-tuning methodologies across all model architectures.

Shifting from hyperparameter search computational costs to performance results, beginning with the performance variations pertaining to the LoRA Alpha and Rank hyperparameters, Figure 7 shows the average evaluation loss, including all model architectures, for each combination of LoRA Rank and Alpha for the LoRA and QLoRA fine-tuning methods. On average, the LoRA fine-tuning method has better performance than the QLoRA fine-tuning method at higher Rank and Alpha, except Rank 64 Alpha 256. Interesting to note is that the average evaluation loss decreases with increasing Rank and increasing Alpha, and peak average performance for a given Rank parameter value occurs when Alpha is maximized. Additionally, peak average performance for any given Rank is achieved when the Alpha parameter value is maximized at four times the Rank value. These observations indicate that, for the QLoRA fine-tuning methods, performance is strongly correlated with increasing Rank and Alpha, and, for QLoRA in particular, increasing the ratio of Alpha to Rank. Furthermore, there is no crossover point observed where increasing Rank, Alpha, and the ratio of Rank to Alpha results in a worse average performance than lower values for those hyperparameters.

The eight best fine-tuned models were ranked in terms of evaluation loss (see Table 3). The model architecture, LoRA Alpha and Rank parameters, if applicable, GPU memory usage, and total training time are also shown. The top-performing model, the full-precision fine-tuned FLAN-T5 Large, utilized the second-least amount of GPU memory during training but required the longest training time. While the LoRA and QLoRA methods are specifically designed to reduce GPU memory consumption, the opposite was observed in Table 3 and Figure 6; the LoRA and QLoRA methods required significantly more memory to train the highest-performing model type, the FLAN-T5 Large, than the full-precision method. The exception to this observed trend is the FLAN-T5 Large model fine-tuned with the LoRA method with a LoRA Rank of 128 and a LoRA Alpha of 512. This model required the least amount of GPU memory to train among the top eight models, with the second-highest training samples processed per second and the second-lowest total training time. An observed trend with the LoRA and QLoRA methods is that the ratio of LoRA Rank to LoRA Alpha among the top-performing models is, with two exceptions, either a 2:1 or 4:1 ratio. Another trend with the LoRA and QLoRA fine-tuned models is that the total training times for the LoRA method are all significantly less than the QLoRA method, while the GPU memory usage for the QLoRA method trends lower than the LoRA method. This trend is consistent with the implementation differences between LoRA and QLoRA, as QLoRA dequantizes the base model during forward and backward passes, incurring additional training time penalties. Also notable is that the top eight models are dominated by the FLAN-T5 Large model architecture, with the FLAN-T5 XL making a sole appearance in third place. Also notable is that the LoRA fine-tuning method appears five times in the top eight, the QLoRA method appears twice, and the full-precision method appears only once.

**Table 3:** Top eight models with lowest evaluation loss.

**Loss**	**Model**	**Method**	**LoRA Alpha**	**LoRA rank**	**GPU usage**	**Training time (s)**
0.06384	FLAN-T5 large	Full-precision	X	X	341	67,419
0.06870	FLAN-T5 large	LoRA	512	128	291	28,680
0.06874	FLAN-T5 XL	QLoRA	256	64	497	31,297
0.06959	FLAN-T5 large	QLoRA	512	128	499	34,208
0.07039	FLAN-T5 large	LoRA	64	64	580	27,745
0.07057	FLAN-T5 large	LoRA	64	128	584	29,116
0.07089	FLAN-T5 large	LoRA	256	64	573	29,317
0.07101	FLAN-T5 large	LoRA	256	128	576	29,582

Fine-tuning method, GPU Memory usage (GB), LoRA Rank and Alpha (if applicable), and Total Training Time (seconds) are shown for each model.

Finally, the results from the ablation study compared the loss obtained when training with and without DSS rationales across model sizes and fine-tuning methods. Table 4 summarizes the results. Across all eight configurations in the ablation study, training with DSS rationales (α = 0.5) yielded lower evaluation loss than label-only training (α = 1.0).

**Table 4:** Ablation study comparing DSS (α = 0.5) vs. label-only training (α = 1.0) across model architectures and fine-tuning methods.

**Model**	**Method**	**Loss (α = 0.5)**	**Loss (α = 1.0)**	**Loss difference**
FLAN-T5 small	Full-precision	0.08826	0.08966	+1.4e-3
FLAN-T5 small	LoRA	0.08247	0.08356	+1.1e-3
FLAN-T5 small	QLoRA	0.08163	0.09758	+1.6e-2
FLAN-T5 base	Full-precision	0.07533	0.08021	+4.9e-3
FLAN-T5 base	LoRA	0.07578	0.07603	+2.5e-4
FLAN-T5 base	QLoRA	0.07739	0.08211	+4.7e-3
FLAN-T5 large	LoRA	0.06870	0.06977	+1.1e-3
FLAN-T5 large	QLoRA	0.06959	0.07326	+3.7e-3

Metrics are averaged over two random seeds.

## 5 Discussion

This work evaluated the performance of three fine-tuning methods—full-precision, LoRA, and QLoRA–across multiple FLAN-T5 model architectures, providing insights into the trade-offs between computational cost, training time, and evaluation performance between those methods. Additionally, the DSS method was utilized to efficiently create a unique dataset utilized for the natural language query to Query DSL translation task, and an ablation study was performed to test the hypothesis of DSS rationales improving model training. Together, these components demonstrate a scalable development pipeline for deploying fine-tuned language models in real-world applications that align and expand on recent advances in efficient fine-tuning methodologies and dataset creation techniques (Hu et al., 2024; Oliver and Wang, 2024; Rajabzadeh et al., 2024).

While the experimental results and ablation findings provide empirical support for the proposed fine-tuning strategy, the broader application is that efficient datasets and efficient computation are directly linked. The following sections interpret the results toward applications in production environments via trade-offs between performance and resource cost, scalability across model sizes and compute budgets, and the specific contribution of DSS rationales to downstream task learning.

### 5.1 Performance trade-offs

The results shown in Table 3 demonstrate clear trade-offs between evaluation performance and GPU memory consumption in fine-tuning methods and model architectures. Although the full-precision fine-tuning method consistently achieved better evaluation loss across the model architectures, with the exception of the FLAN-T5 XL, where resource limitations prevented its employment, it did so at the cost of significantly higher average GPU memory usage for the smaller model architectures (FLAN-T5 Small and FLAN-T5 Base). This result highlights the inherent challenge of employing the full-precision fine-tuning method in resource-constrained environments, where GPU memory is often limited. These GPU memory limitations often prevent, and in this case, did prevent, the application of larger and potentially more capable model architectures to specific problems in environments where GPU memory is a finite resource (Figure 6). This GPU memory cost, however, is offset by the smaller number of training runs required to find the hyperparameter combination that yielded better evaluation performance than the other methods. This offset suggests that while GPU memory-based costs may be a higher up-front cost associated with full-precision fine-tuning, the lower number of runs results in a cheaper total hyperparameter search in terms of total compute time. This demonstrates that for environments where GPU compute capacity is not a constraint and/or environments where time-based usage is a constraint, then full-precision is the most efficient and appropriate method. In practical terms, these findings support a decision framework for selecting a fine-tuning method based on deployment constraints. When peak task performance and shortest model-development cycles are the primary objective, full-precision is the most effective choice. When memory availability is the dominant constraint and model size must be maximized within fixed GPU memory, QLoRA is a suitable option. When both time and GPU memory are constraints, LoRA is the optimal choice. Rather than a single optimal method, the results indicate that environments with varied constraints benefit from different fine-tuning methodology selections.

### 5.2 Scalability

When GPU resources are a primary constraint, the LoRA and QLoRA methods provide memory-efficient alternatives to full-precision fine-tuning for the smaller model architectures like FLAN-T5 Small and FLAN-T5 Base based upon the comparison of evaluation loss performance and memory consumption. When combined with DSS, which reduces the effort required to generate a high-quality dataset for fine-tuning, these methods offer practical solutions for real-world fine-tuning applications where GPU resources are the primary constraint. By reducing dataset creation time, dataset complexity, and memory consumption simultaneously, this pipeline (DSS plus LoRA/QLoRA fine-tuning) provides an opportunity for rapidly deploying fine-tuned, task-specific, language models on memory-constrained devices or smaller cloud configurations.

The memory efficiency of these fine-tuning methods became less evident as model size increased. The LoRA and QLoRA methods required significantly more memory, on average, than the full-precision method when using the FLAN-T5 Large architecture, but the QLoRA method was the only one that enabled the fine-tuning of the FLAN-T5 XL model with the available resources. Given this, the QLoRA method provides an avenue to fine-tune large model architectures on smaller compute clusters and devices at a reasonable performance trade-off to the full-precision method. DSS complements this scalability by enabling the efficient creation of datasets optimized for model fine-tuning, further reducing the total time required to achieve strong performance on domain-specific tasks. In practice, employing the outlined strategy using QLoRA will enable the fine-tuning of large model architectures in memory-constrained environments.

The marginal improvement in QLoRA's training samples per second was observed for the FLAN-T5 XL architecture over the FLAN-T5 Large architecture (Figure 4). This shows that QLoRA's theoretical optimizations over the LoRA method, such as its quantization and paged-memory strategies, provide real-world advantages in computational cost for fine-tuning larger models. This efficiency advantage in samples per second is contrasted by the increased mean training time per run for the QLoRA method. The advantages of requiring less compute are offset by utilizing those resources for longer periods of time.

With regards to the scalability of the hyperparameter search, full-precision fine-tuning included searching over 7 hyperparameters (Table 2) that were baselined according to previous research and only required 3 runs to achieve strong performance. The LoRA and QLoRA methods required searching over an additional 4 hyperparameters (Rank, Alpha, Target Modules, Dropout Rate) that required a large number of runs to converge upon values that enabled comparable performance to the full-precision method.

The analysis of LoRA and QLoRA fine-tuned models revealed a strong correlation between evaluation performance and the values of the LoRA Rank and Alpha hyperparameters. Higher Rank and Alpha values led to improved average evaluation loss, with peak performance observed at ratios of 4:1 for Alpha to Rank. This suggests that increasing both parameters enables the model to learn richer representations without significantly impacting computational efficiency. It is important to note that while this work did not examine Alpha and Rank values that would exceed that 4:1 ratio, there was no indication in the data that increasing Rank and Alpha further would decrease model performance or significantly decrease the computational efficiency of fine-tuning. This suggests that further increases in Rank and Alpha values, and larger ratios of Alpha to Rank, may provide additional boosts to fine-tuning performance. For this reason, this work recommends using a LoRA Alpha to Rank ratio of at least 4:1, with the highest values for Alpha and Rank feasible, as a starting point for hyperparameter searches using these methods.

### 5.3 Effect of DSS rationales on model training

The ablation results in Table 4 were designed to isolate the effect of DSS rationales by comparing training with rationales included (α = 0.5) with training with only labels (α = 1.0) across multiple model sizes and fine-tuning methods. Across the eight configurations examined, training with included DSS rationales yielded lower evaluation loss than label-only training, demonstrating that DSS training consistently improves model fine-tuning on the Query DSL translation task.

The magnitude of the improvement varied as a function of model size and fine-tuning strategy. The largest reduction in evaluation loss occurred in settings where model size and update precision were most limited: FLAN-T5 Small and, in particular, FLAN-T5 Small using QLoRA. In contrast, the smallest performance differences were observed for larger model sizes with higher precision, suggesting that the larger models were able to learn much of the task structure from the prompt and output labels alone. Taken together, these results indicate that rationale supervision acts as a structured input signal that improves learning efficiency for all models and is more impactful when computational constraints impose limits on the size of the student model. Even for larger models, where improvements were smaller, rationales continued to provide measurable improvement, indicating a comprehensive added benefit rather than an effect limited to small model architectures.

These observations align with prior work demonstrating that rationales improve learning efficiency by imposing intermediate reasoning that structures model learning (Hsieh et al., 2023). The present study extends those findings by showing that rationale supervision remains beneficial even when paired with parameter-efficient fine-tuning and quantized model weights on a more complex task, variables that were not present in earlier DSS research. This suggests that DSS rationales are not only a mechanism for improving few-shot generalization but also a tool for accelerating model learning convergence in resource-constrained fine-tuning pipelines.

### 5.4 Comparative analysis of fine-tuning methods

Among the fine-tuning methods, full-precision is shown to be the highest-performing and most computationally efficient method in terms of total time to converge on optimal hyperparameters. These attributes make the combination of DSS and full-precision fine-tuning the most attractive choice for a wide range of applications, particularly in scenarios requiring rapid development cycles or constrained computational budgets. The combination of DSS with the LoRA and QLoRA methods, while less effective in terms of evaluation loss than full-precision, exhibited more efficient GPU memory usage for most model architectures and enabled the training of larger models at the cost of longer training times and a longer total hyperparameter search. These observations make LoRA and QLoRA fine-tuning methods practical options for tasks where memory constraints are a primary consideration and/or where lengthy hyperparameter searches may not be a concern, such as edge deployments, GPU-constrained systems, or limited cloud environments.

Taken together, the performance benchmarking, scalability assessment, and ablation findings indicate that efficiency in dataset construction and efficiency in model fine-tuning accelerate the application of fine-tuned models to specific tasks. DSS reduces the data requirements across all fine-tuning methods, while the constraints of a given resource environment inform the choice between full-precision, LoRA, and QLoRA without altering the underlying training pipeline. From this, the approach introduced in this study can be applied as a modular framework where DSS serves as a consistent foundation for efficient dataset creation, and the fine-tuning method can be selected based on available compute resources, model size, and operational constraints. As a result, the choice of fine-tuning method becomes a deployment decision rather than a performance constraint.

### 5.5 Limitations

Several limitations should be considered when interpreting this work and can be considered directions for future study. First, the empirical evaluation is confined to a single downstream task: translation from natural language questions to Query DSL for a specific downstream data environment. Although this task is representative of a broader family of natural language to formal language translation problems, the conclusions may not directly transfer to other domains, such as open-ended text generation, conversational dialogue, or classification tasks without further validation. Second, the evaluation metrics used in this study are limited. The primary metric is token-level evaluation loss on a held-out validation set, supplemented by computational metrics such as training samples per second, total training time, and GPU memory usage. While useful for characterizing training dynamics and resource consumption, and while all training examples were screened for correctness and validity, the metrics do not directly capture task-level correctness, such as exact match rates on the DSL JSON. Although the ablation shows consistent gains in loss when rationales are included, future work should incorporate additional metrics, such as Bilingual Evaluation Understudy (BLEU), Metric for Evaluation of Translation with Explicit Ordering (METEOR), and Translation Edit Rate (TER) scores, to provide a more complete picture of model performance.

Due to the hyperparameter search and ablation experiments being constrained by finite computational resources, not all combinations of model size and fine-tuning methods could be explored, and therefore, conclusions about scalability are extrapolated from a subset of possible configurations. Similarly, the ablation results are averaged over a limited number of random seeds, which may not fully characterize the variance in training outcomes. Additionally, the methodology focuses on the FLAN-T5 encoder-decoder family and does not include decoder-only architectures that are prevalent in many production LLM deployments. Although the methods presented here are compatible with decoder-only models with only minor code changes, empirical validation on such architectures remains an open direction.

## 6 Conclusions

Large language models are providing large steps forward in the fields of deep learning and natural language processing. In this work, we emphasize the importance of efficiently fine-tuning these models for domain-specific tasks that fall outside of their original training data. The efficiency of the methods used in creating the datasets used for fine-tuning models toward these tasks, and the computational efficiency of the methods used in fine-tuning the models themselves, have significant ramifications on the widespread adoption and use of LLMs in production settings where these models need to be tuned for specific uses. This study provided an efficient strategy for fine-tuning large language models for specific tasks. The combination of the Distilling Step-by-Step method, which enables the rapid creation of fine-tuning datasets that include rationales in addition to labels, and full-precision fine-tuning provides an end-to-end fine-tuning method for rapid development and deployment of task-specific models that match or exceed the performance of larger models on those tasks. Additionally, the comprehensive hyperparameter search and comparative analysis of the fine-tuning methods using the FLAN T5 architecture as an exemplar demonstrate that for resource-constrained environments, DSS with LoRA for fine-tuning, using a LoRA Alpha to Rank ratio of 4:1, balances performance and computation consumption. For environments where GPU compute capacity is a primary concern, utilizing DSS with QLoRA fine-tuning with an Alpha to Rank ratio of 4:1 is a performant strategy. The integration of these methods into production pipelines using a variety of LLM architectures has the potential to significantly decrease the time and cost to produce task-specific models, enabling further integration and research into the utility of these systems.

## Data Availability

The datasets presented in this article are not readily available because dataset utilized in the submission is Controlled Unclassified Information (CUI) from US Department of Defense computer information systems. The methods detailed in the submission are Distribution A. Requests to access the datasets should be directed to benjamin.marsh@usmc.mil.
